# Brain function monitoring during off-pump cardiac surgery: a case report

**DOI:** 10.1186/1757-1626-1-94

**Published:** 2008-08-15

**Authors:** Paolo Zanatta, Enrico Bosco, Piero Di Pasquale, Agarwal Nivedita, Carlo Valfrè, Carlo Sorbara

**Affiliations:** 1 Anesthesia and Intensive Care Department, Treviso Regional Hospital, Piazzale Ospedale n°1, 31100, Treviso, Italy; 2 Anesthesia and Intensive Care Department, Rovigo Hospital, Viale 3 Martiri, 140, 45100, Rovigo, Italy; 3 Neurology Department, Udine University Hospital, Santa Maria della Misericordia, Piazzale Santa Maria della Misericordia, 15 33100, Udine, Italy; 4 Cardiovascular Desease Departement, Treviso Regional Hospital, Piazzale Ospedale n°1, 31100, Treviso, Italy

## Abstract

**Background:**

Early postoperative stroke is an adverse syndrome after coronary bypass surgery. This report focuses on overcoming of cerebral ischemia as a result of haemodynamic instability during heart enucleation in off-pump procedure.

**Case presentation:**

A 67 year old male patient, Caucasian race, with a body mass index of 28, had a recent non-Q posterolateral myocardial infarction one month before and recurrent instable angina. His past history includes an uncontrolled hypertension, dyslipidemia, insulin dependent diabetes mellitus, epiaortic vessel stenosis. The patient was scheduled for an off-pump procedure and monitored with bilateral somatosensory evoked potentials, whose alteration signalled the decrement of the cardiac index during operation.

The somatosensory evoked potentials appeared when the blood pressure was increased with a pharmacological treatment.

**Conclusion:**

During the off-pump coronary bypass surgery, a lower cardiac index, predisposes patients, with multiple stroke risk factors, to a reduction of the cerebral blood flow. Intraoperative somatosensory evoked potentials monitoring provides informations about the functional status of somatosensory cortex to reverse effects of brain ischemia.

## Background

Postoperative stroke is a serious adverse event after coronary artery bypass surgery (CABG) and may be increased in patients with multiple risk factors for cerebral ischemia [1]. The off-pump procedure can reduce neurological complications avoiding the use of cardiopulmonary bypass and aortic manipulation [2,3]. However, this can cause hemodynamic instability related to a low cardiac output, low vascular resistance, preload variation and a physical obstruction of the venous return, with subsequent hypotension [4].

Reduced cerebral perfusion can be further aggravated in patients with significant carotid stenosis [5].

Intraoperative neurophysiological assistance provides information on the brain's functional reserve allowing the anesthesiologist and the surgeon to perform a neuroprotective strategy [6,7].

## Case presentation

A 67 year old male patient, Caucasian race, body mass index of 28, with two-vessel disease not amenable to angioplasty, was scheduled for an off-pump procedure, consisting in a left internal mammary artery graft on anterior descending coronary artery and venous grafts on the obtuse marginal. His medical history included one month before, a non-Q posterolateral myocardial infarction and recurrent instable angina; complete occlusion of the right internal carotid and left vertebral artery and 50% stenosis of the left internal carotid artery; uncontrolled hypertension; dyslipidemia, and insulin dependent diabetes mellitus complicated with lower limb sensory neuropathy. Seven years ago, the patient had suffered a stroke because of closing right carotid artery, without clinical effects.

Brain Magnetic Resonance Imaging Scan diagnosed a suffering circle of Willis. The preoperative echocardiography revealed a mild posterolateral hypokinetic wall movement with normal ejection fraction. The chest X-ray showed moderate aortosclerosis of the ascending aorta. The neurologic examination was negative with the exception of altered tactile sensibility of the legs bilaterally.

During operation we used the Pressure Invasive Continuos Cardiac Output technology  to monitor in continuous, the cardiac output. Epicardial echocardiography was obtained to exclude any atheromatous plaques in the ascending aorta.

SEPs (somatosensory evoked potentials) from median nerve by electrical stimulation were recorded in continuous, after general anesthesia induction. The median nerve were bilaterally stimulated with subdermal needle electrods at the wrist. The recording electrods were placed at the homolateral Erb's point and at the C3'/C4' at opposite side the stimulation site. The stimulation rate was 3.7 Hz. After a baseline obtained with a 300 stimulus average, the ongoing average was obtained with 30 stimulus.

## Results

The SEPs and the hemodynamic parameters did not change until the end of the first graft.

**Figure 1:**
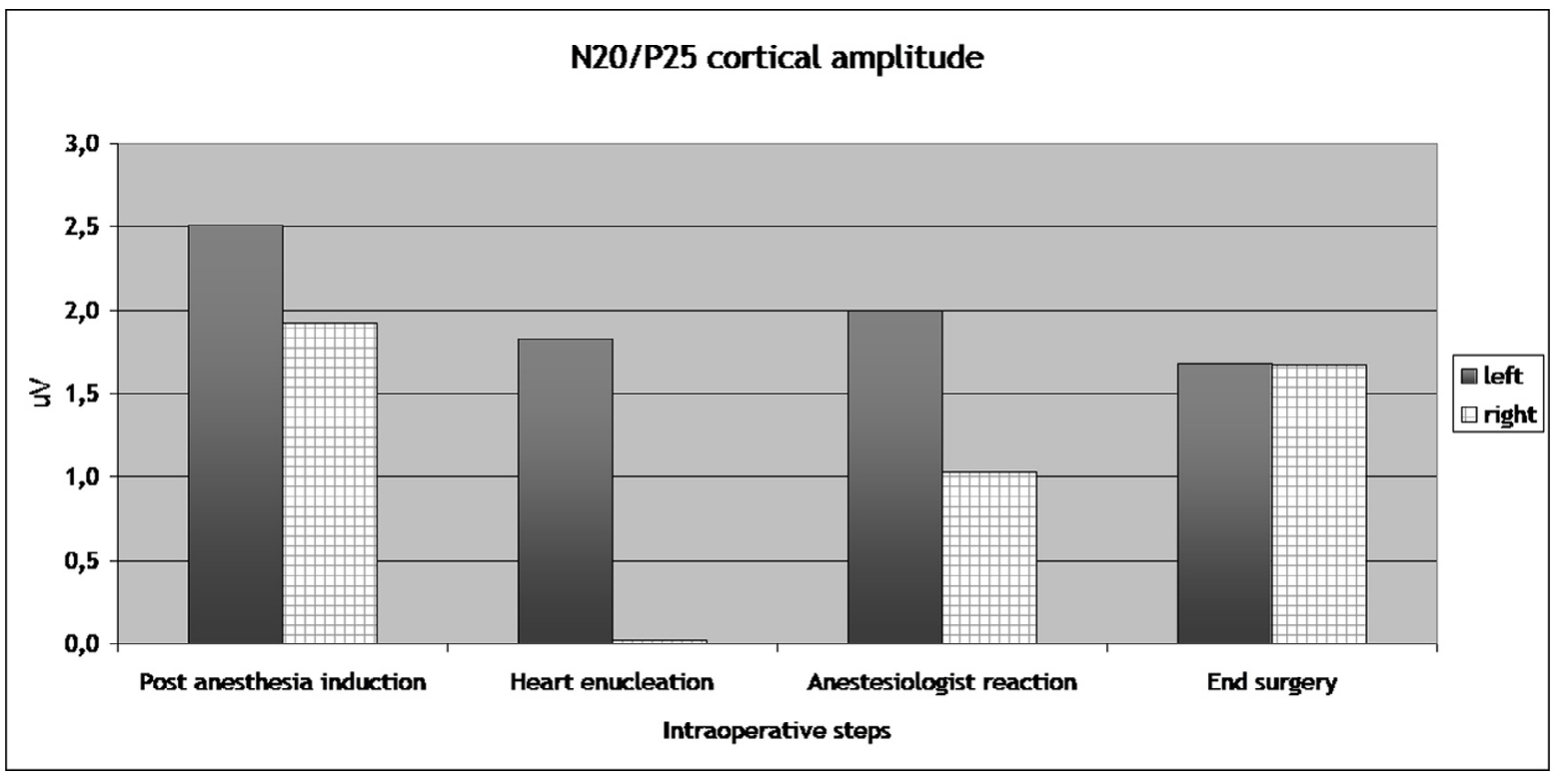
**SEP (N20/P25) amplitude variation during the intraoperative steps**. The right SEP disappeared when the heart is enucleated. Both SEPs have a non significant wave amplitude attenuation at the end of surgery. N20/P25 complex is the most important scalp-recorded cortical component that has a negative peak about 20 msec followed by a positive peak at about 25 msec.

During the heart displacement to perform the second coronary anastomosis, the cardiac index (CI) markedly decreased (from 2.9 to 1.8 l/min/m^2^), without arterial pressure variation and the right SEP disappeared (Table 1), (Figure 1). The left SEP amplitude was reduced by 30%. No variation were noted on the Erb's recording.

**Table 1:** SEP, hemodynamic and respiratory variable recordings and the neuroprotection strategy during the operative steps.

	**Post anaesthesia** ** induction**		**Heart enucleation**		**"Anestesiologist ** **reaction"**		**End surgery**	
	
	Right	Left	Right	Left	Right	Left	Right	Left
N20/P25 latency (msec)	22.67	23.83	-	25.54 ± 0.08	23.86 ± 0.13	25.52 ± 0.08	25.15	23.35
N20/P25 amplitude (uV)	1.922 ± 0.09	2.51 ± 0.13	0.02	1.83 ± 0.007	1.03 ± 0.3	2 ± 0.04	1.67 ± 0.03	1.68 ± 0.01
CF (pulse/min)	80		60		65		65	
SAP (mmHg)	135		130		**173**		146	
MAP (mmHg)	84		91		**114**		95	
DAP (mmHg)	58		70		**76**		67	
CVP (mmHg)	18		24		27		18	
CI (l/min/m^2^)	2.9		**1.8**		**1.8**		2.6	
SVRI (dyn.sec.m^2^/cm^5^)	1820		2977		**3866**		2369	
PaCO_2 _(mmHg)	38		39		40		40.5	
SaO_2 _(mmHg)	100		100		100		100	
T (°C)	36.2		35		34.8		35	

**neuro-protection**								

Norephi (mcg/Kg/h)	-		-		**0.08**		-	
MAC (Et isoflurane)	0.5		0.5		**1**		1	
FiO2 (%)	50		50		**100**		50	
Hb (g/dl)	10		10		**12**		12	
Volume load (liter)					**0.5 HES.+ 0.5 blood**			

N20/P25 = cortical SEP, CF = cardiac frequency, SAP = systolic arterial pressure, MAP = mean arterial pressure, DAP = diastolic arterial pressure, CVP = central venous pressure, CI = cardiac index, SVRI = systemic vascular resistence index, PaCO^2 ^= arterial pressure of CO^2^, SaO^2 ^= oxygen saturation, Norephi = norephinefrine, MAC = minimum alveolar concentration, FiO^2 ^= inspiratory fraction of oxygen, Hb = haemoglobin, T = temperature, HES = Hydroxyethylstarch

After 10 minutes, with establishment of a neuroprotection strategy, the right SEP reappeared (Table 1) (Figure 1). The systolic blood pressure was increased to 173 mmHg using norepinephrine intravenous boluses of 15 mcg. The inspiratory fraction of oxygen was set at 100% until the end of last anastomosis. The volemia was also increased by administering 500 ml of Hydroxyethylstarch and two packed red cells. Furthermore, the brain metabolism was reduced by increased the end tidal minimal alveolar concentration (MAC) of isoflurane until 1. The CI didn't change during this time.

A norepinephrine infusion of 0.08 mcg/Kg/h was then started and maintained until the end of the surgery (Table 1). When the heart was replaced in the pericardium the CI went up to 2.4 l/min/m^2^.

At end surgery the cortical SEP amplitude in the right and left hemispheres was respectively 13% and 33% lower from baseline values which are still in the normal range [7]. No significative variation on the SEP latency were noted during the case.

The patient was estubated after 5 hours without any neurologic impairment.

## Discussion

Cerebral ischemia in off-pump cardiac surgery occurs due to brain hypoperfusion induced by heart dislocation and possible macroembolic events during partial clamping of the aorta. In our patient, the SEP amplitude disappeared after heart enucleation because of reduced brain oxygen delivery. This variation allowed us to increase Cerebral Blood Flow and arterial concentration of oxygen by enhancing the haemoglobin concentration and the inspiratory fraction of oxygen. A bolus of norepinephrine was administered to increase cerebral perfusion pressure. Brain vascular resistances were reduced by increasing the MAC of isoflurane that reduced also the brain oxygen consumption. The increasing dose of volatile agent did not influence the ability to use SEP to monitor the effect of treatment but produced an attenuation of wave amplitude recorded until the end of surgery like few authors have reported [8].

In literature there is no papers about the use of SEPs for monitoring the brain function in high risk patients for cerebral ischemia submitted to off pump cardiac surgery while there is one paper about the use of electroencephalogram in this setting [6].

The SEPs are a reliable method to monitor brain function during surgery and they present some advantages in respect to the Electroencephalogram, because they are particularly resistant to the anaesthesia, moderate hypothermia and enviromental electrical interference because of averaging [7].

We chose SEP because the brain generators of the cortical SEP (N20/P25) are situated within the middle cerebral artery territory which covers 60% of the brain. Thus, continuous monitoring of the cortical SEP not only provides information on the integrity of the Central Nervous System, but also indirectly on the level of cerebral flow necessary to maintain minimal cortical function.

A 50% reduction of the N20 amplitude and a 20% increase in its latency is considered a clear sign of brain ischemia, in absence of ischemic arm, global hypoxia and bolus of anesthetic drugs [7].

## Conclusion

The low cardiac index produced by the heart enucleation during the CABG off-pump, increases the risk of cerebral hypoperfusion. Intraoperative SEP monitoring seems to be a reliable method to perform a neuroprotection strategy and prevent cortical damage also in off pump coronary artery bypass grafting. Further studies are necessary to confirm this hypothesis.

## Abbreviations

CABG: Coronary artery bypass surgery; SEPs: Somatosensory evoked potentials; CI: Cardiac index; MAC: Minimum alveolar concentration; N20/P25: Cortical SEP.

## Competing interests

The authors declare that they have no competing interests.

## Authors' contributions

PZ conceived the work, collected and analyzed the data and write the article. EB analyzed the data. PDP analyzed the data. AN helped to write the article. VC conceived the work and analized the data. SC analized the data. All authors read and approved the final manuscript.

## Consent

Written informed consent was obtained from the patient for publication of this case report and accompanying images. A copy of the written consent is available for review by the Editor-in-Chief of this journal.
